# The LRR receptor Islr2 is required for retinal axon routing at the vertebrate optic chiasm

**DOI:** 10.1186/s13064-015-0050-x

**Published:** 2015-10-22

**Authors:** Paolo Panza, Austen A. Sitko, Hans-Martin Maischein, Iris Koch, Matthias Flötenmeyer, Gavin J. Wright, Kenji Mandai, Carol A. Mason, Christian Söllner

**Affiliations:** Max-Planck-Institut für Entwicklungsbiologie, Abteilung Genetik, Spemannstraße 35, 72076 Tübingen, Germany; Department of Pathology & Cell Biology, Columbia University, College of Physicians and Surgeons, 630 West 168th Street, New York, NY 10032 USA; Department of Neuroscience, Columbia University, College of Physicians and Surgeons, 630 West 168th Street, New York, NY 10032 USA; Present address: Max-Planck-Institut für Herz- und Lungenforschung, Abteilung Genetik der Entwicklung, Ludwigstraße 43, 61231 Bad Nauheim, Germany; Max-Planck-Institut für Entwicklungsbiologie, Elektronenmikroskopie, Spemannstraße 35, 72076 Tübingen, Germany; Wellcome Trust Sanger Institute, Cell Surface Signalling Laboratory, Hinxton, Cambridge, CB10 1HH UK; Department of Biochemistry and Molecular Biology, Kobe University Graduate School of Medicine, 7-5-1 Kusunoki-cho, Chuo-ku, Kobe, Hyogo 650-0017 Japan

**Keywords:** Islr2, Vasorin, Slit-like2, Optic chiasm, Glial knot, Midline crossing, Axon coherence, Zebrafish, Mouse

## Abstract

**Background:**

In the visual system of most binocular vertebrates, the axons of retinal ganglion cells (RGCs) diverge at the diencephalic midline and extend to targets on both ipsi- and contralateral sides of the brain. While a molecular mechanism explaining ipsilateral guidance decisions has been characterized, less is known of how RGC axons cross the midline.

**Results:**

Here, we took advantage of the zebrafish, in which all RGC axons project contralaterally at the optic chiasm, to characterize Islr2 as an RGC receptor required for complete retinal axon midline crossing. We used a systematic extracellular protein-protein interaction screening assay to identify two Vasorin paralogs, Vasna and Vasnb, as specific Islr2 ligands. Antibodies against Vasna and Vasnb reveal cellular populations surrounding the retinal axon pathway, suggesting the involvement of these proteins in guidance decisions made by axons of the optic nerve. Specifically, Vasnb marks the membranes of a cellular barricade located anteriorly to the optic chiasm, a structure termed the “glial knot” in higher vertebrates. Loss of function mutations in either *vasorin* paralog, individually or combined, however, do not exhibit an overt retinal axon projection phenotype, suggesting that additional midline factors, acting either independently or redundantly, compensate for their loss. Analysis of *Islr2* knockout mice supports a scenario in which Islr2 controls the coherence of RGC axons through the ventral midline and optic tract.

**Conclusions:**

Although stereotypic guidance of RGC axons at the vertebrate optic chiasm is controlled by multiple, redundant mechanisms, and despite the differences in ventral diencephalic tissue architecture, we identify a novel role for the LRR receptor Islr2 in ensuring proper axon navigation at the optic chiasm of both zebrafish and mouse.

## Background

The stereotypical trajectory of developing axons is the outcome of chemotactic guidance. However, it also substantially depends on axon extension by local substrate adhesion and axon-axon fasciculation, and regulated passage through signaling landmarks [38]. One of the most clearly defined and best studied choice points encountered *en route* by commissural axons is the embryonic midline. In the vertebrate visual system, retinal ganglion cell (RGCs) axons project into the optic nerve, across the ventral diencephalic midline and into the optic tracts towards their dorsal thalamic and midbrain targets. In most binocular animals, retinal axons approaching the optic chiasm – the crossing point at the midline – diverge into ipsi- and contralateral projections. While the latter is always predominant in size, ipsilaterally-projecting axons vary in number in different organisms, ranging from zero in zebrafish to ~3 % in rodents and ~45 % in primates [25].

*In vitro* studies using retinal explants showed that the cells of the optic chiasm suppress retinal axon growth, irrespective of their laterality of projection [27, 51, 52, 55]. Accordingly, growth cones proceed in a saltatory fashion across the ventral diencephalon and slow down when approaching the midline [20, 22, 32]. In amphibians and mice, a specific population of midline radial glial cells expresses Ephrin-B2, which repels ventro-temporal retinal axons [33, 54]. As a consequence, growth cones expressing the Ephrin-B2 receptor EphB1 abruptly change morphology to turn and ultimately follow the ipsilateral trajectory. Conversely, evidence to date argues that retinal axons with a contralateral trajectory cross the midline by overcoming chemosuppression at the chiasm. In the murine anterior hypothalamus, a cluster of CD44/SSEA1-expressing early neurons is required for retinal axon entry into the chiasm [48], providing the first evidence that the chiasmatic territory is not exclusively refractory to axon growth. Indeed, the expression of Plexin-A1 in these neurons, in conjunction with NrCAM on radial glia, reverses the inhibitory effect of midline-derived Semaphorin-6D, thereby promoting axon growth [27, 53]. Similarly, a VEGF isoform expressed at the mouse optic chiasm acts as an attractant to support crossing of Neuropilin1-positive retinal fibers [15].

In contrast to rodents and primates, fish have an entirely contralateral retinal projection, making it an ideal system in which to dissect mechanisms mediating RGC axon crossing at the midline. In order to identify new molecular and cellular players in this process, we considered that both axon growth and guidance crucially depend on cell-cell and cell-extracellular matrix (ECM) interactions mediated by cell surface and secreted proteins [38]. In this respect, proteins belonging to the leucine-rich repeat (LRR) superfamily – including Slits and Trks – meet important requirements to participate in precise and dynamic processes in neurodevelopment [12]. These molecules display highly specific and dynamic expression patterns, particularly in the nervous system. Cell surface LRR proteins can mediate low affinity interactions with their binding partners. Finally, the number of cell surface LRR proteins is greatly expanded in vertebrates, correlating with the increased complexity of nervous and immune system organization.

Here, by analyzing the spatiotemporal expression patterns of cell surface LRR superfamily members in zebrafish, we identified *islr2* as a LRR receptor-encoding gene expressed in RGCs. Zebrafish larvae mutant for *islr2* aberrantly display ipsilateral retinal projections, demonstrating a role for Islr2 in RGC axon midline crossing. In *Islr2* (alias *Linx*) mutant mice, many retinal axons misproject at and distal to the chiasm, confirming the role of Islr2 in ensuring proper axon routing at this choice point. We also identify two midline factors expressed in the zebrafish optic pathway, Vasna and Vasnb, that bind to Islr2 and analyze the consequences of their loss on the retinal projection.

## Results

### Islr2 is a cell surface LRR-domain-containing receptor expressed by retinal ganglion cells

In order to identify putative novel regulators of midline crossing in zebrafish, we assembled a catalogue of all annotated LRR receptors listed by the zebrafish model organism database, ZFIN [5], starting from the collection of mammalian orthologs for these proteins [13]. Within our dataset of 116 genes, we identified 23 receptors expressed in the zebrafish RGC layer that we hypothesized may play a role in retinal axon guidance (Table 1).

**Table 1:** Zebrafish genes encoding cell surface LRR proteins expressed in RGCs. List of mouse (*Mus musculus*, *Mm*) cell surface LRR genes and their orthologs in zebrafish (*Danio rerio*, *Dr*) displaying expression in RGCs as judged by data available in ZFIN, the zebrafish model organism database

*Mm* gene	Ensembl *Mm* gene ID	*Dr* gene	RGC expression	First stage of expression	Ensembl *Dr* gene ID
*Ntrk2*	ENSMUSG00000055254	*ntrk2b*	y	2 dpf	ENSDARG00000059645
		*ntrk2a*	y	6 dpf	ENSDARG00000059897
*Ntrk3*	ENSMUSG00000059146	*ntrk3a*	y	3 dpf	ENSDARG00000077228
		*ntrk3b*	y	6 dpf	ENSDARG00000063035
*Lrrn1*	ENSMUSG00000034648	*lrrn1*	n		ENSDARG00000060115
		sc:d0413	y	2 dpf	
*Lrrn3*	ENSMUSG00000036295	*lrrn3*	y	2 dpf	ENSDARG00000089923
*Lingo1*	ENSMUSG00000049556	*lingo1a*	y	5 dpf	ENSDARG00000034165
		*lingo1b*	y	2 dpf	ENSDARG00000035899
*Lrrc4*	ENSMUSG00000049939	*lrrc4.1*	y	2 dpf	ENSDARG00000069402
		*lrrc4.2*	n.d.		ENSDARG00000003020
*Islr2*	ENSMUSG00000051243	*islr2*	y	36 hpf	ENSDARG00000051875
*Amigo1*	ENSMUSG00000050947	*amigo1*	y	5 dpf	ENSDARG00000079620
*Lrfn5*	ENSMUSG00000035653	*lrfn5b*	y	2 dpf	ENSDARG00000079396
		*lrfn5a*	n		ENSDARG00000071230
*Flrt1*	ENSMUSG00000047787	*flrt1b*	n		ENSDARG00000075597
		*flrt1a*	y	2 dpf	ENSDARG00000077556
*Lrrtm1*	ENSMUSG00000060780	*lrrtm1*	y	4 dpf	ENSDARG00000052713
*Lrrtm2*	ENSMUSG00000071862	*lrrtm2*	y	4 dpf	ENSDARG00000071374
		*lrrtm2(2)*	n.d.		ENSDARG00000090425
*Lrrtm4*	ENSMUSG00000052581	*lrrtm4l1*	y	2 dpf	ENSDARG00000080015
		*lrrtm4*	n.d.		ENSDARG00000059535
		*lrrtm4(2)*	n.d.		ENSDARG00000078839
		*lrrtm4l2*	y	4 dpf	ENSDARG00000077562
*Lrtm2*	ENSMUSG00000055003	*lrtm2*	y	2 dpf	ENSDARG00000045483
*Slitrk1*	ENSMUSG00000075478	*slitrk1*	y	3 dpf	ENSDARG00000077514
*Slitrk2*	ENSMUSG00000036790	*slitrk2*	y	3 dpf	ENSDARG00000006636
*Slitrk3*	ENSMUSG00000048304	*slitrk3b*	y	3 dpf	ENSDARG00000074739
		*slitrk3a*	n		ENSDARG00000078123
*Slitrk5*	ENSMUSG00000033214	*slitrk5*	y	3 dpf	ENSDARG00000076987
		*slitrk5b*	n		ENSDARG00000074153
*Slitrk6*	ENSMUSG00000045871	*slitrk6*	y	3 dpf	ENSDARG00000087467

**Fig. 1:**
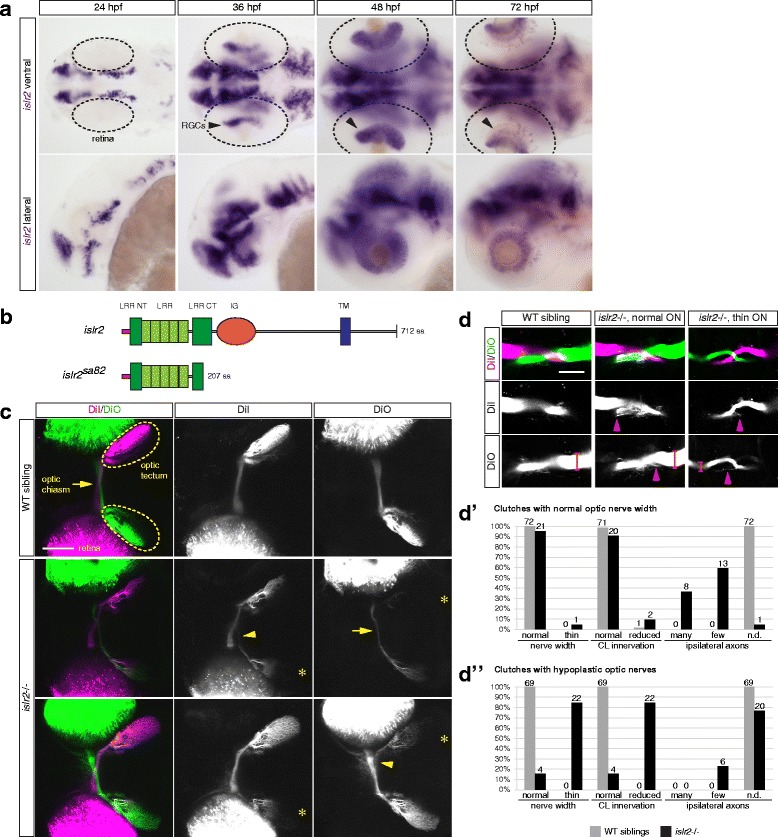
Zebrafish *islr2* is expressed in RGCs and is necessary for complete retinal axon midline crossing. **a** Time-course analysis of *islr2* mRNA expression in zebrafish embryos and larvae. At 36 hpf, the first time point at which the optic chiasm is established, *islr2* is already present in RGCs (arrowheads). At 48 hpf the RGC layer is evenly populated by neurons expressing *islr2*. At 72 hpf we detected a slight decrease in the *islr2* mRNA level and the presence of a subset of *islr2*-positive amacrine cells. Dashed outlines highlight retinas in ventral views. Upper row: ventral view. Lower row: lateral view. Anterior is to the left. **b** Schematic of the *islr2* ^*sa82*^ TILLING allele. The mutant ORF harbors a premature stop codon caused by a C-to-T mismatch, ultimately leading to a truncation of the LRR-CT domain. **c** *. islr2* mutant larvae show ipsilateral retinal projections. First row: dorsal view of a wild-type larva at 5 dpf. The optic nerves from each eye (green and magenta) cross the midline to innervate the contralateral optic tectum (areas delimited by dashed lines). Second and third row: two homozygote mutant animals, displaying ectopic innervation of the ipsilateral tectum (asterisks). The larva in row 2 additionally shows a thinner optic nerve (arrow), while the individual in row 3 has optic nerves with normal width. All pictures are dorsal views and maximum intensity projections of confocal Z-stacks. Anterior is to the left. Scale bar: 50 μm. **d** Direct relationship between optic nerve width (brackets) and ipsilateral misrouting of retinal axons (arrowheads) at the chiasm of *islr2* mutant larvae. Homozygous mutant individuals with normal optic nerve (second column) display a higher number of ipsilateral fibers, compared to mutants with thinner nerves. Single optical sections. Ventral views. Anterior is to the top. ON: optic nerve. Scale bar: 50 μm. **d'** Phenotype distribution in *islr2* mutants compared to siblings. Joint analysis of two clutches of animals showing mostly unaltered optic nerve width. Mutant larvae (black bars) display a higher number of ectopic neurites in the ipsilateral optic tectum, compared to controls (grey bars) and to larvae with thinner optic nerves (compare to d''). The innervation area corresponding to the contralateral optic tectum was unaffected in mutants compared to siblings. CL: contralateral. **d''** Joint analysis of two clutches where larvae with thinner nerves were identified. Only a subset of mutant animals (black bars) displays ipsilateral innervation of the optic tectum, while in many mutants these neurites are undetectable, likely because of the extreme reduction in retinal fiber number. In most mutant animals the innervation of the contralateral optic tectum is reduced, suggesting that thinning of the optic nerve and ipsilateral projections are independent phenotypes. CL: contralateral

Because we are interested in cellular recognition events at the midline, we focused our attention on receptor-encoding genes that are expressed in RGCs between 30 and 36 h, the time between the beginning of RGC differentiation and chiasm establishment. Most genes (e.g. *ntrk2b*, *lrrc4.1*, *flrt1a*, *lrtm2*) were expressed at later stages (48 hpf onwards), leaving a single candidate, *islr2*, fulfilling the temporal expression pattern suitable for further study. In general, *islr2* is expressed in brain nuclei associated with differentiated neurons (Fig. 1a), similar to descriptions of mouse and chick *Islr2* expression [18, 21]. The dynamic pattern of *islr2* transcription and its correlation with the birthdate of RGCs are suggestive of a post-differentiation function held by this protein. Because *islr2* expression starts in the very first cohort of RGCs developing in the retina (Fig. 1a), we hypothesized that Islr2 might be involved in early guidance decisions that these cells take, such as midline crossing.

### Islr2 is necessary for retinal axon midline crossing in zebrafish

To investigate the function of Islr2 *in vivo*, we selected a zebrafish mutant strain harboring a germline mutation in *islr2*, generated by TILLING. The *islr2*^*sa82*^ locus contains a premature nonsense mutation that leads to a protein truncation before the end of the LRR domain (Fig. 1b). As a consequence, this allele is expected to behave as a functional null.

To score for phenotypes related to retinal axon guidance, we anterogradely labeled the optic nerves by intraretinal lipophilic dye injections at 5 dpf, at which time this axon tract is stable, since most error correction events have terminated by 72 hpf [22] and robust visual behaviors are displayed by wild-type larvae [14, 34]. Strikingly, despite being mostly normal morphologically, *islr2* homozygous mutant larvae clearly display ipsilaterally-projecting retinal axons (Fig. 1c, asterisks). We also observed that a subset of *islr2* mutants had thinner optic nerves compared to wild-type siblings (arrow in Fig. 1c, d). The ipsilateral projection phenotype is fully penetrant in larvae with normal optic nerve width, but varies in expressivity, ranging from just a few misprojecting axons to significant innervation of the ipsilateral optic tectum (asterisks in Fig. 1c, d'). In cases where the optic nerve was hypoplastic, the extent of ipsilateral innervation was also reduced (Fig. 1c, d''). This array of phenotypes indicated that the guidance effect of Islr2 could be uncoupled from a putative trophic role of this receptor (described in [30]), which would not, however, be specific for the retinal axon projection. We therefore focused further investigation on the axon pathfinding defects we detected.

**Fig. 2:**
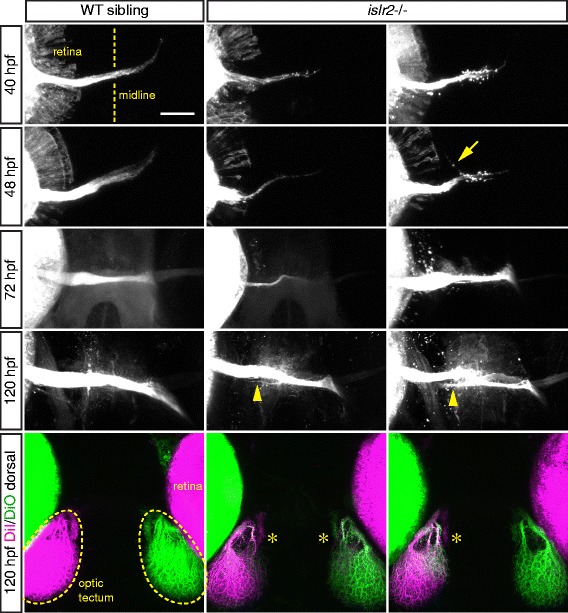
Chronological progression of the *islr2*−/− phenotype. Anterogradely-labeled retinal projections of *islr2* mutant embryos and larvae compared to control siblings. At 40 and 48 hpf growth cones can be observed in the proximity of the midline in mutant embryos, while these seem to have proceeded to the optic tract in wild-type siblings. Additionally, axonal extension defects can be recognized in mutants compared to controls (40 hpf, 48 hpf, 72 hpf, *islr2*−/−, first column). From 48 hpf, ipsilateral neurites (arrow) are visible in mutant embryos and can be traced back to the chiasm. As expected, 5 dpf mutant larvae displayed ectopic innervation of the ipsilateral optic tectum (asterisks). At this stage, *islr2*−/− larvae show misrouted fibers at the chiasm (arrowheads). All pictures are maximum intensity projections of confocal Z-stacks. Anterior is to the top. Scale bar: 50 μm

To characterize the behavior of misprojecting axons during embryonic and early larval development, we labeled the retinal projection of *islr2* mutants at different stages. Mutant axons slightly lag behind the rates of axon extension observed in wild-type siblings at 40 and 48 hpf and growth cones with a spread-out morphology could be recognized in the midline region (Fig. 2). From 48 hpf, it was possible to follow ipsilaterally growing axons from the chiasm (arrow and arrowheads in Fig. 2). Accordingly, 5 dpf larvae from the same clutch presented different levels of ipsilateral innervation of the optic tectum (asterisks in Fig. 2), sometimes associated with optic nerve hypoplasia.

**Fig. 3:**
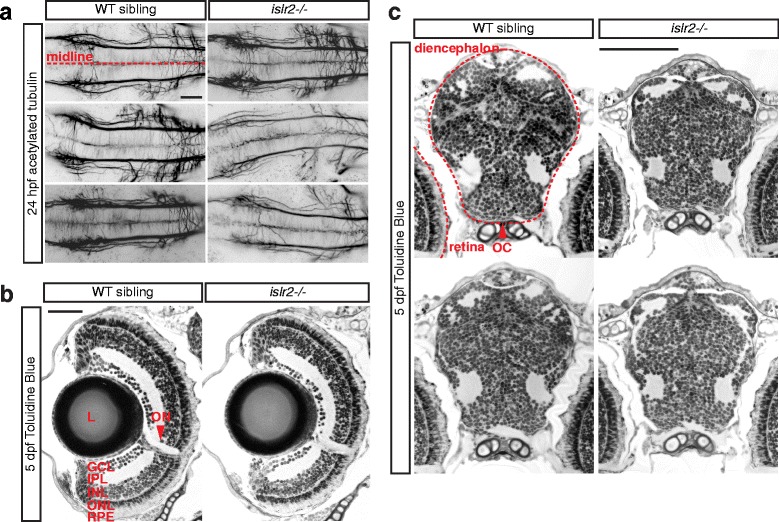
Zebrafish *islr2* mutants do not display widespread axon guidance defects and are morphologically normal. **a** Acetylated tubulin staining of 24 hpf embryos showing central axon tracts at the level of the medial longitudinal fasciculus (MLF). Thinner axon bundles were observed only in a subset of *islr2* mutant animals. The general spacing and directionality of nerve fibers was unaltered compared to controls, indicating absence of guidance defects in these axons. Maximum intensity projections of confocal Z-stacks. Dorsal views. Anterior is to the left. Scale bar: 50 μm. **b** Transverse sections through the retina of 5 dpf *islr2* mutants. Normal size, gross anatomy and layering of the retina were observed in *islr2* mutants. RGC number may be decreased in some *islr2*−/− larvae. L: lens. GCL: ganglion cell layer. IPL: inner plexiform layer. INL: inner nuclear layer. ONL: outer nuclear layer. RPE: retinal pigmented epithelium. ON: optic nerve. Scale bar: 50 μm. **c** Transverse sections at the level of the optic chiasm in *islr2* mutants and siblings. The chiasmatic and ventral diencephalic tissue appears normal in size and patterning, suggesting that the axon guidance phenotype of *islr2* larvae does not originate from mispatterning effects. OC: optic chiasm. Scale bar: 100 μm

In addition to their apparently normal morphology, *islr2* mutant larvae do not present widespread axon guidance defects (Fig. 3a). Sections through the plane of the optic chiasm showed unaffected layered retinal structure (Fig. 3b) and morphologically normal ventral diencephalic tissue (Fig. 3c). Collectively, these data led us to hypothesize that Islr2 function is specifically required in RGCs. Also, because a cohort of axons misproject ipsilaterally at the chiasm in mutants (arrowheads in Fig. 2), we conclude that Islr2 is required for correct midline crossing of RGC axons and not for their guidance to the optic tectum in zebrafish.

### *Islr2* mutant mice display retinal axon defects at the optic chiasm

**Fig. 4:**
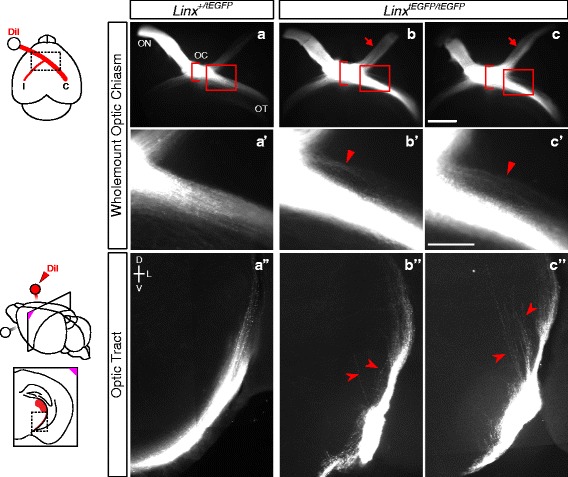
Loss of Islr2 leads to axon routing errors at the optic chiasm and tract in mice. E16.5 *Islr2* mutant mice display expanded chiasms along the antero-posterior axis (brackets in **b** and **c**, compared to **a**). In addition, a greater number of axons enter the opposite optic nerve with retinopetal directionality (arrows in **b** and **c**). Although no clear increase in the number of ipsilaterally-projecting fibers relative to the contralateral projection can be observed, other defects were identified along the postcrossing route of RGC axons. Defasciculation effects in the optic tract were observed both at ventral (**b'** and **c'**)and dorsal locations (**b''** and **c''**, where many axons stray from their normal course (arrowheads). Arrowheads in **b'** and **c'** indicate axons departing rostrally from the chiasm, a common phenotype observed in situations when guidance through the chiasm is impaired. I: ipsilateral. C: contralateral. ON: optic nerve. OC: optic chiasm. OT: optic tract. Scale bars: 500 μm (**a**-**c**), 200 μm (**a'**-**c'** and **a''**-**c''**).

We examined *Islr2* mutant mice (*Linx*^*tEGFP*^ mice; [30]) to further explore the role of Islr2 in the decussation decisions of retinal axons navigating the optic chiasm. Islr2 is expressed by RGCs in the mouse retina ([4] and data not shown), and RGC axons in mutant mice misroute at the optic chiasm and along the optic tract (Fig. 4). A greater number of retinal axons aberrantly re-enter the contralateral optic nerve in *Islr2* mutants (Fig. 4b, c) compared to heterozygous (Fig. 4a) and wild-type (not shown) littermates. The mutant optic chiasm also appears widened rostrocaudally relative to the heterozygote chiasm region (Fig. 4a-c). Although *Islr2* mutant mice do not directly recapitulate the ipsilateral projection phenotype found in zebrafish, there are marked errors in axon routing in both the proximal and distal optic tract in *Islr2* mutants. Bundles of axons defasciculate from the rest of the optic tract on both ipsi- and contralateral sides of the chiasm (Fig. 4b, c, high power of contralateral defasciculation in Fig. 4b', c'), and farther along the tract we detected several axons prematurely leaving the tract and entering the medial thalamus (Fig. 4a''-c''). The common element in the axon routing errors in the mouse and the zebrafish mutants is a lack of coherence of RGC axons, whether at the chiasm or farther along their pathway in the tract. This array of axon routing defects in *Islr2* mutant mice further supports a role for Islr2 in fostering axon bundle coherence and proper tract formation, in response to axon guidance cues at the midline and along the optic tract.

### Zebrafish Islr2 directly interacts with the Vasorin paralogs

To provide a molecular explanation for these effects, we used a systematic protein interaction assay that is designed to detect direct low affinity extracellular protein interactions.

**Fig. 5:**
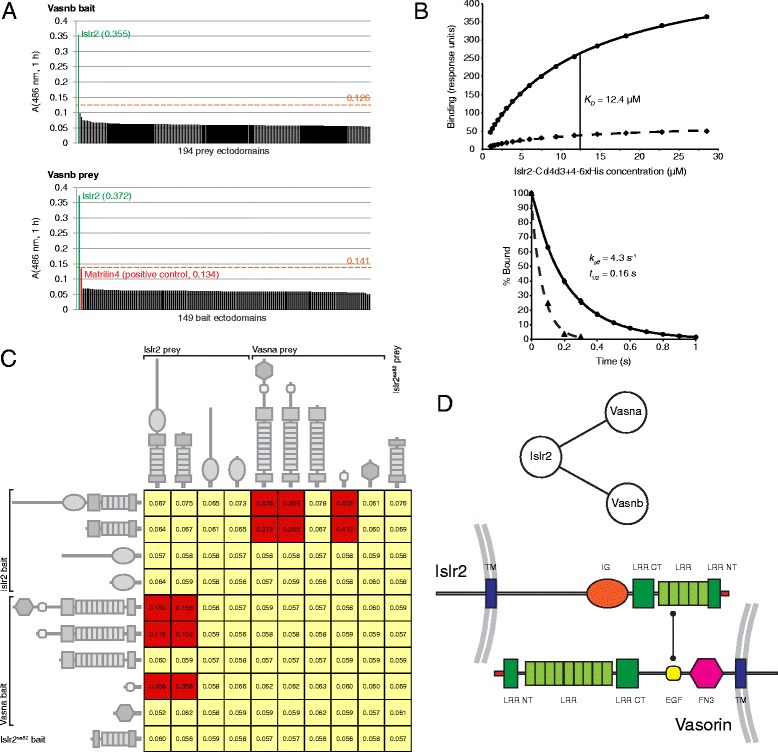
Vasna and Vasnb specifically and directly bind Islr2 with low affinity. **a**. Identification of the Vasnb-Islr2 interaction using the AVEXIS assay. Vasnb interacted with Islr2 and no other protein when presented either as bait against 194 prey ectodomains (upper panel) or as prey against 149 baits (lower panel). Statistical threshold (dashed lines) is calculated as 3 standard deviations over the mean. **b**. Quantification of the interaction between Vasna and Islr2 using surface plasmon resonance. Upper panel: Serial dilutions of purified monomeric Islr2-Cd4d3+4-6xHis were sequentially injected until binding equilibrium was reached over flow cells in which biotinylated Vasna ectodomains were immobilized on a streptavidin-coated sensor chip at high (solid line) and low (dashed line) levels. An equilibrium binding constant (*K*_D_) of 12.4 μM was calculated by non-linear curve fitting to both binding curves. Lower panel: kinetic analysis of the Vasna-Islr2 interaction. A dissociation rate constant (*k*_off_) was calculated by globally fitting a first order decay curve to the dissociation phase of three concentrations (7 μM, 14 μM, 28 μM) of Islr2-Cd4d3+4-6xHis; the interaction half-life (t_1/2_) was calculated as t_1/2_ = ln2/*k*_off_. Each data point represents average values from normalized dissociation data (error bars = ± 1 standard deviation, *n* = 3). Control dissociation measurements (dashed line) are from 28 μM Islr2-Cd4d3+4-6xHis injected over a flow cell containing the Cd4d3+4 tag alone. **c**. AVEXIS screening to identify the extracellular domains mediating the interaction between Vasna and Islr2. We observed nitrocefin hydrolysis whenever the EGF domain of Vasna could bind to the LRR domain of Islr2. Conversely, the truncated LRR domain encoded by *islr2* ^*sa82*^ did not show activity, indicating that the zebrafish mutant allele is functionally null. **d**. Schematic model for the interaction between Vasorin and Islr2, where the proteins bind *in trans*, a property suggested by the non-overlapping expression patterns of the two genes

Using AVEXIS [6] we had previously determined that Vasna is an Islr2 ligand [47]. By screening an expanded library totaling 194 zebrafish receptor ectodomains [17], we confirmed the Islr2-Vasna interaction and additionally detected specific binding with its paralog, Vasnb (Fig. 5a). No other interactions with Islr2, or with the two Vasorin paralogs were identified. Both Islr2-Vasna and Islr2-Vasnb interactions were detected in both prey/bait orientations, demonstrating the specificity of the observed binding events (Fig. 5a; [17]).

We validated and quantified the interaction between zebrafish Islr2 and Vasna by measuring the equilibrium binding affinity and kinetic parameters using surface plasmon resonance (SPR). The ectodomain of Islr2 was purified and resolved as a monodisperse peak using gel filtration (data not shown). The equilibrium dissociation constant was calculated by injecting serial dilutions of monomeric purified Islr2 over a surface containing immobilized Vasna using a BIAcore instrument. Clear saturable binding was observed, from which an equilibrium dissociation constant (*K*_*D*_) of 12.4 μM was calculated by nonlinear curve fitting to a Langmuir binding isotherm (Fig. 5b, upper panel). This equilibrium measurement agreed well with independently-determined kinetic parameters (*k*_off_ = 4.3 s^−1^), corresponding to a half-life of 0.16 s (Fig. 5b, lower panel). The transient nature of this interaction is consistent with other cell surface receptor interactions when measured in their monomeric state [50].

### The LRR domain of Islr2 interacts with the EGF domain of Vasorin

The extracellular protein architectures of Islr2 and Vasorin consist of a series of functional domains that typically mediate protein-protein interactions, such as the LRR module. We therefore expected that discrete protein domains would be involved in the physical binding between Islr2 and Vasorin. To determine where the interaction interface was located on the two proteins, single- and two- domain truncation constructs were generated and tested for binding using AVEXIS. We observed that the LRR domain of Islr2 bound to the small EGF domain of Vasna (Fig. 5c).

Several proteins represented in our library also contain EGF domains. Islr2, however, could not bind to these proteins, suggesting that Islr2 specifically recognizes the EGF domain in Vasorin.

Because the LRR domain of Islr2 is sufficient to mediate the interaction with Vasorin, we confirmed that the protein that would be produced from the *islr2*^*sa82*^ allele could not bind Vasorin (Fig. 5c), demonstrating that *islr2*^*sa82*^ produces an inactive LRR domain and it is functionally null.

### The Vasorin paralogs are expressed along the zebrafish retinal axon pathway

**Fig. 6:**
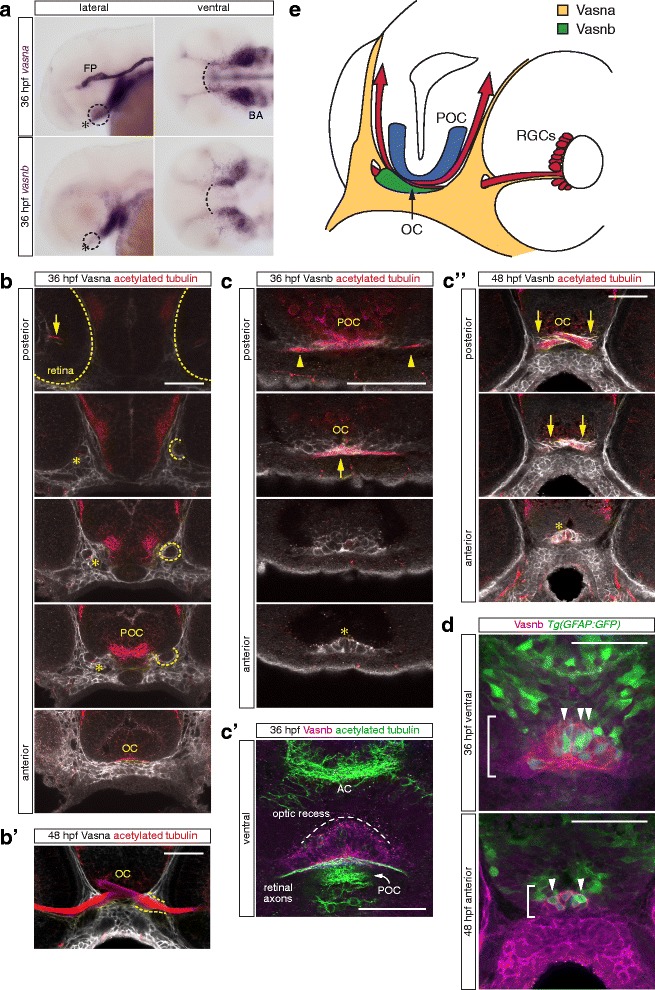
Vasna and Vasnb are specifically expressed in the retinal axon pathway at 36 and 48 hpf. **a** Whole-mount *in situ* hybridization of *vasna* and *vasnb* mRNA reveals the expression of these genes (dashed circles) in the region of the RGC axon commissure (asterisk). At 36 hpf, when the optic chiasm is first established, *vasna* is highly expressed in the floor plate (FP) and prospective branchial arches (BA). Additionally, *vasna* marks mesenchymal tissue associated with the retinal axon pathway. *vasnb* expression appears to border anteriorly the *vasna*-positive ventral domain (dashed arc). *vasnb* mRNA level is relatively low in this region. Lateral and ventral views. Anterior is to the left. FP: floor plate. BA: branchial arches. **b** A polyclonal anti-Vasna antibody shows Vasna localization to the membrane of mesenchymal cells surrounding ventro-laterally the retinal projection. At 36 hpf, Vasna-positive cells of the primitive optic artery (arrow) are already associated to retinal axons in the eye (dashed outlines). These two cell types stay in contact until retinal axons enter the diencephalic floor. At this point, axons lose contact with the vasculature and encounter the opening of a tunneling structure, marked by Vasna on its surface (dashed circles). Axons always associate with the ventral side of this channel (asterisks). At its exit, retinal axons are brought in the neighborhood of the prospective optic chiasm, which is bordered ventrally by Vasna-positive mesenchymal tissue. Each picture is a single optical section from a Z-stack encompassing antero-posteriorly the whole course of retinal axons. Frontal views. POC: postoptic commissure. OC: optic chiasm. Scale bar: 50 μm. **b'** At 48 hpf, many axons have extended through the lateral Vasna-positive tunnels (dashed lines). Qualitatively the expression pattern of Vasna does not change from 36 hpf. Frontal view. OC: optic chiasm. Scale bar: 50 μm. **c** A polyclonal antibody against Vasnb marks a subset of midline cells in the ventral diencephalon. As soon as retinal axons enter the diencephalon, they are in contact with laterally-positioned Vasnb-positive cells (arrowheads). At the chiasmatic midline (arrow), retinal axons are surrounded by Vasnb-positive membranes. Vasnb-expressing cells populate the anterior-most ventral diencephalic tissue, until the forebrain ventricle opens. This location is consistent with the reported position of the glial knot (asterisk). Single optical sections through the ventral diencephalon, posterior to anterior, are shown. Frontal views. POC: postoptic commissure. OC: optic chiasm. Scale bar: 50 μm. **c'** Ventral view of an embryo stained for Vasnb and acetylated tubulin. The preoptic area, between the optic recess and the chiasm is populated by cells expressing Vasnb. Vasnb-positive cells enwrap retinal axons. The picture is a Z-projection of 4 consecutive optical sections. Anterior is to the top. AC: anterior commissure. POC: postoptic commissure. Scale bar: 50 μm. **c''** At 48 hpf, Vasnb is still detected on cells associated to the optic nerves (arrows) and in the glial knot (asterisk). From this stage Vasnb is clearly located on membranes wrapping the optic nerves in ventral positions along their course inside the brain. Each picture is a single optical section through the preoptic area. Frontal views. OC: optic chiasm. Scale bar: 50 μm. **d** A subset of Vasnb-positive cells in the knot region (brackets) expresses the transgenic glial marker *Tg(gfap:GFP)* (arrowheads). Single optical sections from confocal Z-stacks. Ventral and anterior views. Scale bars: 50 μm. **e** Tridimensional model of the retinal projection in zebrafish embryos at about 36 hpf. Retinal axons (red) are always in contact with Vasna-positive cells (yellow). Their ventral diencephalic course is shaped anteriorly by the Vasnb expression domain (green). The POC is shown in blue and borders the RGC axons caudally. OC: optic chiasm. POC: postoptic commissure. RGCs: retinal ganglion cells

With the aim of characterizing the site of expression of Islr2 ligands, we performed mRNA *in situ* hybridization in embryos at different stages of development. *vasna* is highly expressed in the floor plate, starting at about 12 hpf [7]. From 36 hpf, *vasna* transcripts progressively become restricted to the anterior portion of the floor plate, while appearing in the branchial arches primordia and head mesenchyme (Fig. 6a). *vasnb* has a more restricted expression pattern and is localized to the ventral diencephalic tissue neighboring early embryonic commissures, namely the anterior and postoptic commissure, before 36 hpf. In addition, weak expression was detected at later stages in the region of the branchial arches (Fig. 6a). In the central nervous system, *islr2* did not appear to co-localize with *vasna* or *vasnb*. The respective expression domains were mostly adjacent (data not shown and [47]), suggesting that the corresponding proteins may interact *in trans* (Fig. 5d).

We raised polyclonal antibodies against the extracellular portion of Vasna and Vasnb to determine where the corresponding proteins are localized along the retinotectal axon pathway. Vasna and Vasnb were both detected from 36 hpf in the ventral diencephalic region, precisely where retinal axons pioneer the optic nerve. In accordance with our *in situ* hybridization analysis, Vasna labels the membrane of lateral mesenchymal tissue of the embryonic head, surrounding the optic stalks and, ventrally, the chiasm. Weak Vasna staining was already present on the membranes of the primitive optic artery, close to the site of early RGC differentiation (arrow in Fig. 6b). From there, retinal axons grow in direct contact with the retinal vasculature to reach the interface between the retina and the diencephalon, which is marked by a sheet of Vasna-positive mesenchymal tissue. Vasna staining reveals the border of a hollow structure, most likely the lumen of the optic stalk, which channels axons from the retina to the diencephalon, orienting them toward the chiasm (dashed circles in Fig. 6b). Because this structure is present at early stages, when only a single axon is growing into the brain, it constitutes a prepatterned route which may guide pioneering axons to the chiasm. At 36 hpf, only a few axons grew inside this channeling structure, maintaining contact with its ventral-most Vasna-positive membrane (asterisks in Fig. 6b), but by 48 hpf many more were added, so that essentially the entire channel was filled with growing axons (Fig. 6b'). It appears that the postcrossing route of retinal axons is also marked by Vasna-positive tissue, in particular along the optic tract.

Vasnb was detected from 36 hpf in the anterior-most portion of the ventral diencephalic midline. Such a restricted location precisely encompasses the tissue between the optic recess (anterior/dorsal), which outlines the diencephalic ventricular area, and the optic chiasm (located posteriorly/ventrally) (Fig. 6c, c'). Retinal axons at the chiasm course through Vasnb-positive tissue (arrows in Fig. 6c, c''). At 48 hpf, the lining of the optic nerves along their ventral diencephalic route shows immunoreactivity for Vasnb (Fig. 6c''). In addition, both at 36 and 48 hpf, a restricted cellular population located anteriorly to the chiasm is marked by Vasnb antibodies (asterisk in Fig. 6c, c''). The position of this cellular array is strikingly reminiscent of that of the “glial knot” in mouse and chicken [43, 46]. Vasnb expression appears to be absent from the GFAP-positive commissural glial bridges that have been described to facilitate anterior and postoptic commissural (POC) axon extension [1]. However, double staining of Vasnb together with a *gfap:GFP* transgenic insertion [3] revealed that some Vasnb-positive cells also express this glial marker (arrowheads in Fig. 6d), consistent with immunostains of the knot in cat brains [44].

Collectively, the spatiotemporal restriction of Vasnb together with the fact that Vasna-positive tissues border the complete path of retinal axons and that retinal axons course through the caudal face of the anteriorly-positioned Vasnb cells indicate that Vasorin ligands could be key factors responsible for Islr2 function in retinal axon midline crossing.

### Vasorin-deficient zebrafish do not exhibit overt RGC axon guidance phenotypes

**Fig. 7:**
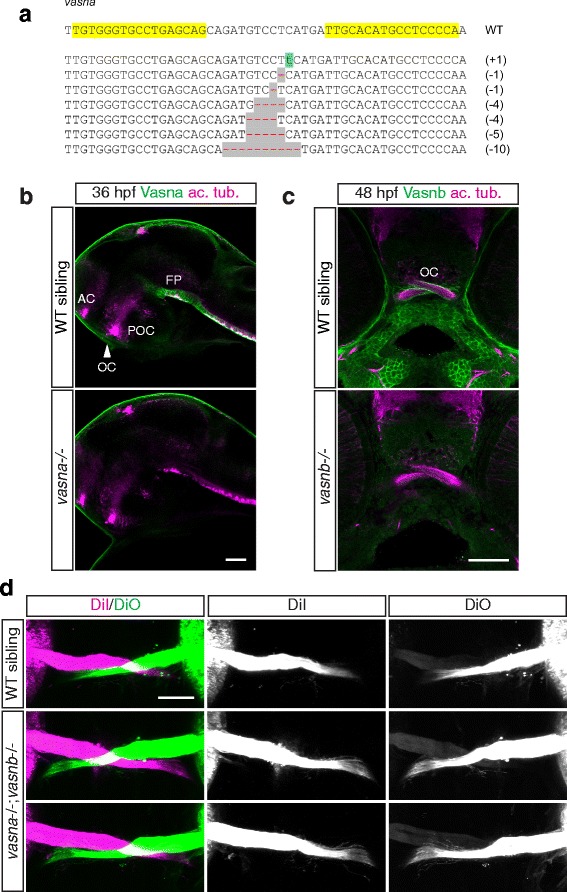
*vasna/vasnb* double mutants do not display overt axon guidance defects at the optic chiasm. **a** Alleles generated by targeted mutagenesis of the *vasna* locus, using TALEN technology. All alleles are followed by premature stop codons, leading to the translation of a short and functionally inactive protein. Yellow highlights show the genomic DNA target sequence for the engineered nucleases. **b** *vasna* alleles generated by targeted mutagenesis display complete loss of Vasna protein in homozygosity. AC: anterior commissure. FP: floor plate. OC: optic chiasm. POC: postoptic commissure. Single optical sections from confocal Z-stacks. Lateral views. Anterior is to the left. Scale bar: 50 μm. **c** The *vasnb* ^*sa6027*^ TILLING allele leads to complete loss of function, as suggested by the absence of Vasnb immunoreactivity. Single optical sections from confocal Z-stacks. Frontal views. Scale bar: 50 μm. **d** 5 dpf *vasna/vasnb* double mutant larvae do not display strong axon guidance phenotypes at the optic chiasm. Ventral views of anterogradely-labeled optic nerves at the optic chiasm. Anterior is to the top. Scale bar: 50 μm

Given the direct physical interaction between Islr2 and the Vasorin paralogs and their expression patterns within the visual system, we investigated whether zebrafish larvae homozygous for Vasorin loss of function alleles exhibited retinal axon pathfinding defects. By injecting TALENs directed to the very beginning of the *vasna* coding sequence, we generated insertions and deletions causing frameshifts and, consequently, premature stop codons (Fig. 7a). Homozygous *vasna* mutants were viable and fertile, and displayed no morphological phenotype to adulthood.

We confirmed that the *vasna* mutant alleles did not produce Vasna protein by immunostaining. Despite the complete lack of Vasna expression (Fig. 7b), we observed no major axonal phenotype in the brain commissures of *vasna* mutants. In addition, mutant larvae analyzed at 5 dpf by DiI/DiO injections in the retina did not display erratic guidance or abnormal number of retinal axons (data not shown). Because Vasnb expression is restricted to midline tissue anterior to the chiasm, we hypothesized that it was a good candidate to mediate Islr2 function at the midline; however, no retinotectal projection phenotype was observed in a *vasnb*^*sa6027*^ TILLING allele. Nonetheless, as in the case of *vasna* mutant larvae, the Vasnb protein was not detected in mutant embryos (Fig. 7c). Given the possibility that the Vasorin paralogs could functionally substitute for each other even if not co-expressed, similarly to Slit1 and Slit2 in mouse [37], we bred larvae that were doubly defective in both *vasna* and *vasnb*. These individuals also exhibited normal decussation of the retinal projection (Fig. 7d) and appeared morphologically normal throughout larval development, suggesting the existence of additional factors compensating for their loss.

## Discussion

Cell surface and secreted proteins are fundamental mediators of axon guidance, especially because they dictate how growth cones interface with the environment during navigation. The identification of novel receptor-ligand pairs controlling axon directionality, however, is complicated by the existence of accessory molecules that modify the interactions between cognate receptors and ligands and by the transiency of interactions mediated by these molecules. This typical feature of membrane proteins is essential for regulating dynamic functions during nervous system development, but obfuscates both the identification of related phenotypes and their recapitulation *ex vivo*. In the context of retinal axon pathfinding in zebrafish, even unbiased discovery strategies, such as large-scale forward genetic screens, have resulted in the isolation of very few guidance receptors acting cell-autonomously in misrouted axons [26, 49]. In fact, the majority of mutants showing defects in the laterality of the retinal projection also display mispatterning of the ventral midline tissue, suggesting that local tissue architecture in the ventral diencephalon is critical for the actuation of midline crossing [1, 2, 41]. Therefore, genetic approaches to dissect redundant biological processes suffer from intrinsic limitations and might require the utilization of sensitized mutant backgrounds.

Here we have used the zebrafish as a model for the decision of RGC axons to cross the midline, a process common to the ontogeny of the visual pathway in all animals [25]. To overcome the constraints of genetic analysis mentioned above, we took a candidate approach by screening the zebrafish repertoire of cell surface LRR molecules, known modulators of axon pathfinding. We spatiotemporally compared the available gene expression patterns with the steps in axon growth leading to the formation of the retinal projection in zebrafish. The expression dynamics of our final candidate, *islr2*, correlate with the timing of RGC generation and chiasm establishment. *Islr2* knockout mice display striking neuronal defects, including thinning and increased branching of sensory and motor limb projections [30] and complete absence of the internal capsule [31]. However, while *Islr2* expression in RGCs is shared in zebrafish, chick [18], and mouse [4], nothing was known about the functional role of *Islr2* in the visual system. Here we demonstrate that Islr2 controls axon routing at the zebrafish and mouse optic chiasm.

Axons of both the optic nerve and of central longitudinal tracts can appear decreased in number in zebrafish *islr2* mutants. To understand whether this phenotype is due to disrupted intraretinal axon guidance, we used an RGC-specific *isl2b:GFP* transgenic line [36] and did not detect ectopic neurites in mutant retinas (data not shown). As an alternative explanation, newly differentiated RGCs could be susceptible to higher rates of apoptosis in mutant embryos. In agreement with results from mouse *Islr2* mutants [30], *islr2*/*tp53* double mutants do not rescue *islr2*−/− defects in fish (data not shown). These results leave lack of trophic support as a candidate explanation for the optic nerve hypoplasia observed in *islr2* mutants. Although consistent with a described role of Islr2 in modulating neurotrophin and GDNF/Ret signaling [30], this phenotype was not fully penetrant.

The discovery that Vasorin proteins bind to Islr2 made them first-order candidates to modulate retinal axon navigation at the midline. Indeed, the expression patterns of Vasna and Vasnb in conjunction with axonal labeling revealed a striking association of positive cells with retinal axons, a finding that emphasizes the predictive power of our protein-protein interaction screening approach.

Vasorin was first described to directly bind TGF-beta [23], perhaps acting as a secreted inhibitor after ADAM17-mediated cleavage [29]. By employing novel antibody reagents, we detected confined Vasorin immunoreactivity to the cell membrane and did not observe long-range shedding of the antigen from its source.

Importantly, the close apposition of retinal axons to Vasorin-positive cells and their misrouting in *islr2* mutants introduced the question of how Islr2-Vasorin interactions may control axon pathfinding. RGC axons closely follow Vasna-positive cells, extending from the retina up to the contralateral optic tract. Moreover, the physical interaction between Islr2 and Vasorin is very transient. These results suggest that Vasorin may not function as an axon repellant, but rather constitutes a permissive, contact-dependent signal, one that may require another molecular player. Whereas the phenotype of zebrafish mutants indicates that Islr2 is involved in the decision to cross the midline, the defects observed in mouse mutants rather suggest that Islr2 could more generally control axonal coherence along the chiasm and tract. A defect in fasciculation may explain the slower advance and stalling of growth cones in zebrafish *islr2* mutants, as well as ipsilateral growth. Indeed, it is not yet clear how axon bundle integrity may affect guidance decisions [37]. We hypothesize that axonal organization could directly depend on interactions with the surrounding tissue, for which Vasorin is an outstanding candidate.

In contrast to its paralog, Vasnb marks fewer and more localized midline cells at stages when the optic chiasm first forms. Similar strategically-located cellular formations have been found to border various commissural axon tracts in the vertebrate brain. These associations control axon directionality at choice points, as – for instance – in the corpus callosum, a commissure which requires a transient midline cell population (the “glial sling”) for appropriate crossing of inter-cortical projections [42, 44, 45]. In zebrafish, a GFAP-positive bridge-like formation is patterned by Hedgehog and Slit signals and provides the physical substrate for POC axon growth [1]. These glial cells, however, are located just posteriorly to the optic chiasm, whereas Vasnb-positive cells surround axons and form a rostral keel.

A histological description of the murine E13.5 optic chiasm [43] revealed the selective association of early retinal fibers that cross the midline with a cellular population anterior to the chiasm, the “glial knot”. Because of its morphology (a compact cellular array with short, interwoven protrusions and little ECM), the knot was thought to act as a barrier for retinal axons, which never penetrate this barricade and are deflected instead. However, axons keep contact with cells of the knot, suggesting that they are not strongly repelled. Such a realignment effect was proposed to derive from a combination of repulsion and adhesion.

As RGC axons that are crossing the midline in both zebrafish and mouse physically associate with morphologically distinct cells anterior to the optic chiasm, it is possible that cells of the knot direct the fasciculation of retinal axons, constricting the bundle of axons and thereby directing their growth across the midline. Because knot-like cells appear to be conserved in fish, chick [46], mouse [43] and cat [44], it will be important in the future to determine the role of the glial knot in the formation of the contralateral retinal projection. Here, we identified Vasnb as a marker for the knot cells in fish.

Available genetic data indicate that the retinal axon trajectory is controlled at the murine optic chiasm by multiple functionally overlapping systems, thus generating redundancy [15, 27, 37, 54]. In both mouse and zebrafish, removal of components that control the transit of axons through the chiasm cannot completely prevent axon extension. Additionally, in zebrafish – in which all retinal axons cross the midline – mutants displaying ipsilateral projections in the context of normal brain patterning do so only in an incompletely penetrant manner [11, 19, 39]. In sharp contrast, *islr2* mutant larvae display a fully penetrant ipsilateral projection when optic nerve thickness is normal and exhibit unaltered patterning of the ventral diencephalon as judged by staining for Vasnb (data not shown). *vasna*/*vasnb* double knockouts, however, fail to recapitulate defects found in *islr2* mutants. In the light of a redundant signaling environment, it is not surprising that transient low affinity interactions can be compensated for during axon guidance. We therefore propose that at least a third player is capable of masking Vasorin loss-of-function in zebrafish, probably by acting through Islr2.

Proteoglycans of the heparan sulfate (HSPGs) and chondroitin sulfate (CSPGs) families are plausible candidates for modulating Islr2-Vasorin signaling. Both molecules are expressed in the murine retina, optic chiasm midline and optic tract [8, 9]. Mouse mutants in the key enzyme for HS production, *EXT1*, display retinopetal innervation to the contralateral optic nerve, a feature strikingly reminiscent of *Islr2* mutants, as well as *Slit1*/*Slit2* double knockouts [37]. EXT1 was found to cooperate with Slit2 in ensuring appropriate routing of retinal fibers at the chiasm [24]. Zebrafish *extl3*/*ext2* double mutants also lack axon coherence at the chiasm and show ectopic retinopetal innervation. In addition, expression of HSPGs in zebrafish embryos is intimately associated with the retinal axon pathway and closely resembles the localization of Vasna [28]. Collectively, these data suggest that Islr2 may cooperate with Slit signaling and proteoglycans to control axon fasciculation and growth through the chiasmatic region: loss of axon bundle integrity may determine the axon extension defects observed in *islr2* zebrafish mutants and lead to expansion of the optic chiasm in *Linx* mice.

## Conclusions

Here we implicate a novel LRR receptor, Islr2, in retinal axon guidance in zebrafish and mouse. *Islr2* larvae with normally populated optic nerves display an ectopic ipsilateral retinal projection originating from the chiasm. In addition, *Islr2* mutant mice present a similar phenotype of straying axons caudal to the midline, misrouting of axons into the opposite optic nerve, and manifest a widening of the chiasm. These results reveal a function for Islr2 in controlling RGC axon guidance and coherence through the optic chiasm, in conjunction with trophic support in the retina, across species.

One of the zebrafish Islr2 binding partners, Vasnb, is specifically expressed by cells anterior to the optic chiasm. We hypothesize that these cells belong to the glial knot and mediate midline crossing of zebrafish retinal axons along the ventral diencephalon, as they are the primary cell type contacted by RGC axons extending through the chiasm. These results highlight the redundant signaling environment at the optic chiasm, orchestrating axon guidance choices to cross or avoid the midline, all the while maintaining coherent bundles as axons project to their targets.

## Methods

### Transgenic lines and animal maintenance

All zebrafish experiments were performed in accordance with the guidelines of the Max Planck Society and approved by the Regierungspräsidium Tübingen (Aktenzeichen: 35/9185.46). The following zebrafish lines were used and reared as described previously [35]: wild-type Tübingen strain, *islr2*^*sa82*^ and *vasnb*^*sa6027*^ (Zebrafish Mutation Project, Wellcome Trust Sanger Institute, Hinxton, UK), *vasna*, *Tg(−17.6isl2b:GFP)*^*zc7Tg*^ [36], *Tg(gfap:GFP)*^*mi2001Tg*^ [3].

The *Linx*^*tEGFP*^ mouse line [30] was maintained on a C57BL/6 background. The mutant and control samples were prepared from the same litter. The day when a vaginal plug was observed was designated as embryonic day (E) 0.5. All animal experiments were performed in accordance with the institutional guidelines and approved by the administrative panel on laboratory animal care of Kobe University. The protocol was approved by the Committee on the Ethics of Animal Experiments of Kobe University Graduate School of Medicine (Permit Number: P120206).

### *In situ* hybridization

Single-gene expression detection was performed according to Filippi et al. [16]. Two-color fluorescent *in situ* hybridization followed the protocol of Clay and Ramakrishnan [10].

### Anterograde labeling of retinal axons with lipophilic dyes

5 dpf zebrafish larvae were fixed in 4 % formaldehyde overnight at 4 °C. Larvae were extensively washed at room temperature in distilled water prior to injections. Retinal injections were performed on agarose injection plates, with larvae fitted in the grooves. DiI, DiO and DiD (V-22885, V-22886, V-22887, Molecular Probes) were pressure-injected in the RGC layer until completely filled. Injected larvae were kept in distilled water in the dark overnight at room temperature and imaged directly.

For earlier time points, embryos and larvae were fixed in 4 % formaldehyde at room temperature for 1 h and then overnight at 4 °C.

DiI was used to anterogradely label the retinal axons from one eye in *Linx*^*tEGFP*^ embryos. Embryonic heads of mice of either sex were fixed in 4 % PFA overnight, then rinsed with PBS. The right eyecup was removed from the head and small crystals of DiI were placed onto the optic disc and pushed into the optic nerve head with a fine glass micropipette. The eye was placed back into the optic cup and heads were incubated at 37 °C in 4 % PFA for 7 days. Following incubation, brains were dissected from heads and imaged as intact wholemounts under a stereomicroscope. The brains were then embedded in 3 % agarose, and serial 100 μm-thick vibratome sections were collected and mounted for imaging.

### Histological methods

Fish larvae were fixed in 100 mM PO_4_ buffer (pH 7.2) containing 4 % formaldehyde and 2.5 % glutaraldehyde at room temperature for 2 h. Further fixation was carried out overnight at 4 °C. The samples were post-fixed with 0.1 % osmium tetroxide on ice for one hour and then stained with 1 % aqueous uranyl acetate at 4 °C for one hour in the dark. The samples were dehydrated in a graded series of ethanol/water concentrations; subsequently, a graded series of Epon/araldite resin (EMS Araldite 502/Embed 812 Kit) in propylene oxide was used for embedding.

Three micrometer semi-thin transverse sections were stained with Toluidine Blue and embedded in Epon before imaging at 16x magnification on a Zeiss Axioplan widefield microscope equipped with a F-View camera (Soft Imaging Systems).

### AVEXIS

Protein interaction screening was performed according to Bushell et al. [6]. Briefly, recombinant protein ectodomains were expressed in HEK293-6E cells and supernatants were used in the interaction assay. Prey protein conformation was internally controlled using zebrafish Matrilin4 as bait to detect the prey pentamerization domain derived from Cartilage Oligomeric Matrix Protein (COMP). Bait proteins were captured on streptavidin-coated 96-well plates and assayed for binding following incubation with normalized prey proteins for 1 h at room temperature. In order to reveal binding events, 50 μl of 100 μg/ml nitrocefin (a beta-lactamase substrate; 484400, Calbiochem) was added. 486 nm absorbance values were measured after 1 h incubation in the dark at room temperature using a μQuant spectrophotometer (Bio-Tek Instruments).

### Protein purification and BIAcore analysis

Protein purification and BIAcore analysis was performed essentially as described [6]. Briefly, the entire ectodomain of Islr2 was produced in mammalian cells as a Cd4d3+4-6xHis-tagged protein and purified on a 1 ml HisTrap column (GE Healthcare). Protein aggregates, which are known to influence kinetic experiments, were removed by gel filtration using a 125 ml Superose6 column prior to BIAcore analysis. Vasna-Cd4d3+4-bio baits were immobilized onto a streptavidin-coated sensor chip and approximate molar equivalents of Cd4d3+4-bio were used as a reference. All binding studies were performed in HBS-EP buffer (BIAcore) at zebrafish physiological temperature (28 °C). Flow rates of 100 μl/min were used for kinetic studies to minimize rebinding effects and 20 μl/min for equilibrium studies, and data were collected at 10 Hz. Off-rate constants and equilibrium dissociation constants were calculated using non-linear curve fits to the data in the BIAevaluation software.

### Antibody generation

Vasna and Vasnb ectodomains fused to Cd4d3+4-6xHis were expressed in HEK293E cells. Supernatants were collected and purified using HisTrap HP columns (GE Healthcare). During the first immunization, a mixture of 1 ml antigen (0.5 mg/ml) and 1 ml Complete Freund’s Adjuvant (Sigma-Aldrich) was injected in rabbits. At 14 days intervals, boost injections were performed. 1 ml antigen (0.2 mg/ml) mixed with 1 ml Incomplete Freund’s Adjuvant (Sigma-Aldrich) was used for the first boost. Two additional boosts using 1 ml antigen (0.1 mg/ml) mixed with 1 ml Incomplete Freund’s Adjuvant were performed. All injections were directed subcutaneously and intramuscularly. 50 ml bleeds were taken starting from 1 week after the last boost. These antibodies were used as crude sera.

### Immunohistochemistry

Staged animals were manually freed from chorions if necessary and fixed in 4 % formaldehyde overnight at 4 °C. The samples were washed in PBS and dehydrated through a methanol series. The embryos were stored in 100 % methanol at −20 °C until used.

All embryos were permeabilized in cold acetone at −20 °C and washed again in 100 % methanol. After rehydration by scalar washes in methanol dilutions, embryos were digested using Proteinase K (10 μg/ml) and subsequently fixed in 4 % formaldehyde for 20 min at room temperature. A blocking step with 10 % normal goat serum (NGS, Sigma-Aldrich) was carried out for 5–6 h at room temperature. Primary antibodies were added at a 1:1000 dilution in 10 % NGS overnight at 4 °C. The samples were washed in PBST (PBS, 0.1 % Tween20) and incubated with secondary antibodies (1:250 in 1 % NGS) overnight at 4 °C, protected from light. After several PBST washes, the samples were post-fixed and brought in glycerol through scalar washes.

The primary antibodies used in this study are: mouse anti-acetylated tubulin (clone 6-11B-1, T6793, Sigma-Aldrich), anti-Vasna, anti-Vasnb. Secondary antibodies used: goat anti-mouse Alexa647 (A21236, Molecular Probes), goat anti-rabbit Alexa488 (A11008, Molecular Probes).

### Generation of *vasna* mutants

DNA constructs containing TALENs directed against the *vasna* locus were purchased from Cellectis Bioresearch. Briefly, mRNA was *in vitro* transcribed using the mMessage mMachine Kit and purified using the MEGAclear Kit (both Ambion). One-cell stage zebrafish embryos were injected with 2–4 nl of a solution containing 50 or 75 ng/μl of each TALEN. Adult F_0_ fish were incrossed and F_1_ fish were genotyped and used to retrieve single alleles by outcrossing. Genotyping primers were: 5’-ACCAGAGGTGTCCAATCCTG-3’ (forward) and 5’-TCCACAAAGTCCTGCTGTTG-3’ (reverse). *vasna* mutants analyzed in this work are transallelic combinations.

### Image acquisition and processing

Images were acquired on a Zeiss LSM 510 Meta or on a Zeiss LSM 780 NLO confocal microscope using a 25x glycerol-immersed objective. Injected larvae were mounted in 3 % methylcellulose, jaws were surgically removed. Collected scans were processed using Fiji [40], by generating a maximum intensity projection of the stacks, unless otherwise specified.

Wholemount images of the optic chiasm of *Linx*^*tEGFP*^ mice were acquired with a Leica 165 fluorescent stereomicroscope using a Plan Apo 1.0X objective with 5X and 12X zoom, and Leica Application Suite V4.2 software. Images of coronal sections were acquired on a Zeiss AxioImager M2 microscope with an AxioCam MRm camera, using Neurolucida software (V11.03, MicroBrightField Systems), with a Fluar 5X objective lens.
